# Profiling compliers and noncompliers for instrumental variable analysis with covariates: A weighting approach

**DOI:** 10.1371/journal.pone.0283223

**Published:** 2023-06-15

**Authors:** Byeong Yeob Choi

**Affiliations:** Department of Population Health Sciences, UT Health San Antonio, San Antonio, TX, United States of America; Yale University, UNITED STATES

## Abstract

Instrumental variable (IV) estimation is an essential tool to estimate the causal effect of a treatment in randomized experiments when noncompliance exists. In such studies, standard statistical approaches can be biased because compliers and noncompliers can differ in unmeasured ways that affect both the compliance behavior and outcome. Based on a few assumptions such as monotonicity, the IV estimand represents the causal effect of compliers. Profiling compliers and noncompliers has important implications because the IV estimand applies only to compliers. A method for estimating the covariate means for compliers and noncompliers has recently been proposed in political sciences literature. However, this approach requires an assumption that the instrument is randomly assigned, which confines the application of this approach to randomized experiments. In this study, we present two weighting methods for profiling compliers and noncompliers when the instrument and compliance behavior are confounded by several covariates. The proposed approach can be used for both experimental and nonexperimental studies, and hence is more broadly applicable. For the development, an instrumental propensity score is adopted to account for confounded instruments. We demonstrate the utility of the proposed methods based on simulation and real data experiments.

## Introduction

Instrumental variable (IV) analysis is used to estimate the causal effect of a treatment in the presence of unmeasured confounders. Angrist et al. [1] demonstrated that when the treatment assignment is randomized, the IV estimand represents the average causal effect of compliers, who comply with the assigned treatment. This estimand is called the local average treatment effect (LATE). Marbach and Hangartner [2] presented identification results to profile compliers and noncompliers, the required assumptions, and the estimators. Their work is important because the LATE addresses only the causal effect on the subpopulation of compliers, but not on the whole population. We will refer to their approach as the MH approach.

The MH approach is useful for studying the profiles of compliers and noncompliers with unconfounded instruments. However, instruments are often confounded, which means that they are random only if several confounders are controlled. For example, the IV analysis of Abadie [3] studied the effects of 401(k) participation on savings, and family income was controlled in the IV model to make 401(k) eligibility become a valid IV. Card [4] used two-stage least squares (2SLS) to estimate the effect of education on earnings, where IV was an indicator of whether an individual grew up near a 4-year college. His 2SLS model included some covariates such as age, race, and family backgrounds to make college proximity a valid IV.

Before the MH approach, few researchers devised methods for profiling compliers with confounded instruments. For example, Baiocchi et al. [5] identified the complier mean as a ratio of two quantities, similar to our approach. However, their approach involves integrating a conditional compliance probability with respect to the conditional joint probability of all measured covariates, given the covariate to be profiled. Thus, a practical method to perform this integration calculation is needed to apply the result. Hangartner et al. [6] used matching to control for IV confounding and applied the MH approach based on that the IV is approximately as-if randomized in the matched data set. However, matching does not guarantee a complete removal of covariate imbalance particularly when there are many matching factors.

After the landmark papers of Imbens and Angrist [7], and Angrist, Imbens, and Rubin [1], several researchers developed IV methods with covariates. For example, Abadie [3], Frolich [8], and Tan [9] presented IV methods based on regression, matching, and weighting for adjustments of the confounders of the instrument. These authors commonly adopted an instrumental propensity score (IPS), which is the probability of being encouraged toward the active treatment conditional on measured covariates, to identify the characteristics of compliers. In this study, we adopt the IPS to develop a weighting approach that is more generally applicable than the MH approach. Our approach is based on inverse probability weighting (IPW) and augmented IPW (AIPW).

The remainder of this article is organized as follows. We briefly review the MH approach, and then present our weighting approach that accounts for confounded IVs. Based on simulations, we evaluate the proposed IPW and AIPW methods in data with confounded IVs by some covariates. The practical utility of the proposed methods is illustrated by a study of education on future earnings. We end the article with concluding remarks.

## Profiling approach for unconfounded instruments

We introduce some notations and assumptions to define the profiles of compliers and noncompliers. Let *Z* ∈ {0, 1} be the binary instrument, with 1 indicating encouragement toward treatment and 0 indicating encouragement toward control. Let *D*(*z*) ∈ {0, 1} be the binary potential treatment value that would be observed if *Z* = *z*. The observed treatment is defined as *D* = *ZD*(1) + (1 − *Z*)*D*(0). We define a column vector of covariates *X*. To refer to the variables for subject *i* among *N* subjects, we add a subscript “*i*” to these notations. In other words, {*Z*_*i*_, *D*_*i*_(0), *D*_*i*_(1), *X*_*i*_} denote the instrument, potential treatment values, and vector of covariates for subject *i*, where *X*_*i*_ = (*X*_*i*1_, …, *X*_*ip*_)^*T*^ and *X*_*ij*_ is the *j*th covariate for subject *i*.

Angrist et al. [1] classified the population into four groups based on the values of *D*(1) and *D*(0): compliers if *D*(1) > *D*(0), always-takers if *D*(1) = *D*(0) = 1, never-takers if *D*(1) = *D*(0) = 0, and defiers if *D*(1) < *D*(0). Because defiers are excluded under Assumption 1 below, noncompliers in IV analysis include only the always-takers and never-takers.

Marbach and Hangartner [2] presented two assumptions to identify the profiles of compliers and noncompliers for IV analysis:

**Assumption 1** *(Monotonicity) D*(1) > *D*(0).

**Assumption 2** *(Independence of the instrument)* {*D*(0), *D*(1), *X*} ⊥ *Z*.

Assumption 1 excludes defiers. Assumption 2 means that *Z* is randomly assigned without controlling any covariates, which occurs in randomized experiments. Based on Assumptions 1 and 2, Marbach and Hangartner [2] identified the covariate means for always-takers, never-takers, and compliers. However, these identification results are only applicable when Assumption 2 is satisfied. The results of Marbach and Hangartner [2] imply that the means of *X*_*ij*_ for always-takers and never-takers can be identified by

$$\begin{array}{ccc} E[X_{ij}|D_{i}\left(1\right)=D_{i}\left(0\right)=1] & = & E[X_{ij}|D_{i}=1,Z_{i}=0],\\ E[X_{ij}|D_{i}\left(1\right)=D_{i}\left(0\right)=0] & = & E[X_{ij}|D_{i}=0,Z_{i}=1]. \end{array}$$

The covariate mean for compliers can be identified by

$$\begin{array}{cc} & E[X_{ij}|D_{i}\left(1\right)>D_{i}\left(0\right)]=\\ & \frac{E\left[X_{ij}\right]-E\left[X_{ij}|D_{i}=1,Z_{i}=0\right]P\left[D_{i}=1|Z_{i}=0\right]-E\left[X_{ij}|D_{i}=0,Z_{i}=1\right]P\left[D_{i}=0|Z_{i}=1\right]}{E\left[D_{i}|Z_{i}=1\right]-E\left[D_{i}|Z_{i}=0\right]}. \end{array}$$

The approach of Marbach and Hangartner [2] can be directly extended to the cases with confounded instruments when the covariates to be profiled are distinct from those that must be controlled to satisfy Assumption 2. Suppose that there exists a vector of covariates, *W*, such that Assumption 2 holds if this vector is adjusted for. In other words, {*D*_*i*_(0), *D*_*i*_(1), *X*_*ij*_} ⊥ *Z*_*i*_|*W*_*i*_. Then, the means of *X*_*ij*_ for always-takers and never-takers are identified by

$$\begin{array}{ccc} E[X_{ij}|D_{i}\left(1\right)=D_{i}\left(0\right)=1,W_{i}] & = & E[X_{ij}|D_{i}=1,Z_{i}=0,W_{i}],\\ E[X_{ij}|D_{i}\left(1\right)=D_{i}\left(0\right)=0,W_{i}] & = & E[X_{ij}|D_{i}=0,Z_{i}=1,W_{i}]. \end{array}$$

Clearly, these equations are not much helpful if *W*_*i*_ contains *X*_*ij*_. Based on these equations, the mean of *X*_*ij*_ for compliers can be identified by adding *W*_*i*_ to the equation as conditioning variables: *E*[*X*_*ij*_|*D*_*i*_(1) > *D*_*i*_(0), *W*_*i*_]. However, this extension has very limited applicability, which provides motivation for our weighting approach.

## Weighting approach for confounded instruments

In this section, we propose two weighting methods to identify the profiles of compliers and noncompliers when the IV and potential treatment values are confounded by several observed covariates.

### Inverse probability weighting (IPW)

To develop an IPW method, we first replace Assumption 2 with the following, which is adopted from Abadie [3], Frolich [8], and Tan [9]:

**Assumption 3** *(Conditional independence of the instrument)* {*D*(0), *D*(1)} ⊥ *Z*|*X*.

Assumption 3 means that the instrument is independent of the potential treatment variables conditional on *X*. Therefore, *Z* and {*D*(0), *D*(1)} can be confounded by *X*. We address this confounding by adopting an IPS, *e*(*X*) = *P*[*Z* = 1|*X*]. The IPSs are unknown unless the study is a randomized experiment. In addition to Assumption 3, we impose an assumption that the IPS for every subject is strictly between 0 and 1.

**Assumption 4** *(Positivity)* 0 < *e*(*X*) < 1 for every subject.

We introduce additional notations as follows. Let *f*_*j*_(*x*) = *P*[*X*_*ij*_ = *x*] represent the probability mass function or probability density function of *X*_*ij*_. To refer to the densities of *X*_*j*_ for compliers, always-takers, and never-takers, we add superscripts “*c*”, “*a*”, and “*n*” to *f*_*j*_(*x*), respectively. In other words, these densities are denoted by

$$\begin{array}{ccc} f_{j}^{c}\left(x\right) & = & P[X_{ij}=x|D_{i}\left(1\right)>D_{i}\left(0\right)],\\ f_{j}^{a}\left(x\right) & = & P[X_{ij}=x|D_{i}\left(1\right)=D_{i}\left(0\right)=1],\\ f_{j}^{n}\left(x\right) & = & P[X_{ij}=x|D_{i}\left(1\right)=D_{i}\left(0\right)=0]. \end{array}$$

The complier mean of *X*_*ij*_ can be expressed as

$$\begin{array}{c} \mu_{j}^{c}=E\left[X_{ij}|D_{i}\left(1\right)>D_{i}\left(0\right)\right]=\int xf_{j}^{c}\left(x\right)dx. \end{array}$$

By Bayes’ theorem,

$$\begin{array}{c} f_{j}^{c}\left(x\right)=P\left[D_{i}\left(1\right)>D_{i}\left(0\right)|X_{ij}=x\right]f_{j}\left(x\right)/P\left[D_{i}\left(1\right)>D_{i}\left(0\right)\right]. \end{array}$$

Therefore, $\mu_{j}^{c}$ can be written as

$$\begin{array}{c} \mu_{j}^{c}=\frac{\int xP\left[D_{i}\left(1\right)>D_{i}\left(0\right)|X_{ij}=x\right]f_{j}\left(x\right)dx}{P[D_{i}\left(1\right)>D_{i}\left(0\right)]}. \end{array}$$ (1)

Under monotonicity, the denominator of Eq (1) can be written as *P*[*D*_*i*_(1) > *D*_*i*_(0)] = *E*[*D*_*i*_(1) − *D*_*i*_(0)]. This proportion of compliers can be identified by *E*[*D*_*i*_(1) − *D*_*i*_(0)] = *E*[*D*_*i*_|*Z*_*i*_ = 1] − *E*[*D*_*i*_|*Z*_*i*_ = 0] if Assumption 2 holds. However, when the instrument is confounded by *X*, this identification result does not hold because *E*[*D*_*i*_(*z*)] ≠ *E*[*D*_*i*_|*Z*_*i*_ = *z*] for *z* = 0, 1. To identify *E*[*D*_*i*_(1) − *D*_*i*_(0)], we express the expectation of each potential treatment as the IPW mean based on the result of Lunceford and Davidian [10]:

$$\begin{array}{c} E\left[D_{i}\left(1\right)\right]=E[\frac{Z_{i}D_{i}}{e_{i}}],\;E\left[D_{i}\left(0\right)\right]=E[\frac{\left(1-Z_{i}\right)D_{i}}{1-e_{i}}]. \end{array}$$

Note that under monotonicity,

$$\begin{array}{c} P\left[D_{i}\left(1\right)>D_{i}\left(0\right)|X_{ij}=x\right]=E\left[D_{i}\left(1\right)-D_{i}\left(0\right)|X_{ij}=x\right]. \end{array}$$

Hence, the numerator of Eq (1) can be written as

$$\begin{array}{c} \int E\left[x\left\{D_{i}\left(1\right)-D_{i}\left(0\right)\right\}|X_{ij}=x\right]f_{j}\left(x\right)dx=E\left[X_{ij}D_{i}\left(1\right)\right]-E\left[X_{ij}D_{i}\left(0\right)\right], \end{array}$$

which represents the average causal effect of *Z*_*i*_ on *X*_*ij*_*D*_*i*_. As a result, $\mu_{j}^{c}$ can be expressed as

$$\begin{array}{c} \mu_{j}^{c}=\frac{E\left[X_{ij}D_{i}\left(1\right)\right]-E\left[X_{ij}D_{i}\left(0\right)\right]}{E\left[D_{i}\left(1\right)\right]-E\left[D_{i}\left(0\right)\right]}. \end{array}$$

To identify *E*[*X*_*ij*_*D*_*i*_(1)], it is worthwhile to note that *e*_*i*_ = *E*[*Z*_*i*_|*D*_*i*_(1), *X*_*i*_] because *Z*_*i*_ and *D*_*i*_(1) are independent conditional on *X*_*i*_ under Assumption 3. Then, it follows that

$$\begin{array}{c} E\left[X_{ij}D_{i}\left(1\right)\right]=E[\frac{X_{ij}D_{i}\left(1\right)}{e_{i}}E\left[Z_{i}|D_{i}\left(1\right),X_{i}\right]]=E[E[\frac{X_{ij}D_{i}\left(1\right)Z_{i}}{e_{i}}|D_{i}\left(1\right),X_{i}]] \end{array}$$ (2)

Based on that *D*_*i*_(1)*Z*_*i*_ = *D*_*i*_*Z*_*i*_ and the law of total expectation, Eq (2) becomes

$$\begin{array}{c} E\left[X_{ij}D_{i}\left(1\right)\right]=E[\frac{Z_{i}D_{i}X_{ij}}{e_{i}}]. \end{array}$$ (3)

In a similar way, *E*[*X*_*ij*_*D*_*i*_(0)] can be identified by

$$\begin{array}{c} E\left[X_{ij}D_{i}\left(0\right)\right]=E[\frac{\left(1-Z_{i}\right)D_{i}X_{ij}}{1-e_{i}}]. \end{array}$$ (4)

Therefore, $\mu_{j}^{c}$ can be identified as described below in Theorem 1, which summarizes the nonparametric identification results for profiling compliers and noncompliers via IPW. The means of *X*_*j*_ for always-takers and never-takers are defined as

$$\begin{array}{ccc} \mu_{j}^{a} & = & E\left[X_{ij}|D_{i}\left(1\right)=D_{i}\left(0\right)=1\right]=\int xf_{j}^{a}\left(x\right)dx,\\ \mu_{j}^{n} & = & E\left[X_{ij}|D_{i}\left(1\right)=D_{i}\left(0\right)=0\right]=\int xf_{j}^{n}\left(x\right)dx. \end{array}$$

The proofs for the IPW representations of $\mu_{j}^{a}$ and $\mu_{j}^{n}$ are provided in Appendix A.

**Theorem 1** *(IPW representations) Under Assumptions 1, 3 and 4, the means of X*_*ij*_ *for compliers, always-takers, and never-takers are identified by the following IPW representations, respectively*:

$$\begin{array}{ccc} \mu_{j}^{c} & = & \frac{E[\frac{Z_{i}D_{i}X_{ij}}{e_{i}}]-E[\frac{\left(1-Z_{i}\right)D_{i}X_{ij}}{1-e_{i}}]}{E[\frac{Z_{i}D_{i}}{e_{i}}]-E[\frac{\left(1-Z_{i}\right)D_{i}}{1-e_{i}}]},\\ \mu_{j}^{a} & = & \frac{E[\frac{\left(1-Z_{i}\right)D_{i}X_{ij}}{1-e_{i}}]}{E[\frac{\left(1-Z_{i}\right)D_{i}}{1-e_{i}}]},\\ \mu_{j}^{n} & = & \frac{E[\frac{Z_{i}\left(1-D_{i}\right)X_{ij}}{e_{i}}]}{E[\frac{Z_{i}\left(1-D_{i}\right)}{e_{i}}]}. \end{array}$$

For estimation, we assume that the IPS is parameterized with a regression parameter vector *β*: *e*_*i*_ = *e*(*X*_*i*_, *β*). In practice, we use a logistic regression model to estimate *e*(*X*_*i*_, *β*):

$$\begin{array}{c} e\left(X_{i},\beta \right)=\frac{\text{exp}(X_{i}^{T}\beta )}{1+\text{exp}(X_{i}^{T}\beta )}. \end{array}$$

The parameter *β* is estimated by maximum likelihood (ML). Let $\hat{\beta }$ denotes the ML estimator. Then, Theorem 1 suggests immediately the following IPW estimators:

$$\begin{array}{ccc} {\hat{\mu }}_{j}^{c}\left(IPW\right) & = & \frac{(\sum_{i=1}^{N}\frac{Z_{i}}{{\hat{e}}_{i}}{)}^{-1}\sum_{i=1}^{N}\frac{Z_{i}D_{i}X_{ij}}{{\hat{e}}_{i}}-(\sum_{i=1}^{N}\frac{1-Z_{i}}{1-{\hat{e}}_{i}}{)}^{-1}\sum_{i=1}^{N}\frac{\left(1-Z_{i}\right)D_{i}X_{ij}}{1-{\hat{e}}_{i}}}{(\sum_{i=1}^{N}\frac{Z_{i}}{{\hat{e}}_{i}}{)}^{-1}\sum_{i=1}^{N}\frac{Z_{i}D_{i}}{{\hat{e}}_{i}}-(\sum_{i=1}^{N}\frac{1-Z_{i}}{1-{\hat{e}}_{i}}{)}^{-1}\sum_{i=1}^{N}\frac{\left(1-Z_{i}\right)D_{i}}{1-{\hat{e}}_{i}}},\\ {\hat{\mu }}_{j}^{a}\left(IPW\right) & = & \frac{\sum_{i=1}^{N}\frac{\left(1-Z_{i}\right)D_{i}X_{ij}}{1-{\hat{e}}_{i}}}{\sum_{i=1}^{N}\frac{\left(1-Z_{i}\right)D_{i}}{1-{\hat{e}}_{i}}},\\ {\hat{\mu }}_{j}^{n}\left(IPW\right) & = & \frac{\sum_{i=1}^{N}\frac{Z_{i}\left(1-D_{i}\right)X_{ij}}{{\hat{e}}_{i}}}{\sum_{i=1}^{N}\frac{Z_{i}\left(1-D_{i}\right)}{{\hat{e}}_{i}}}, \end{array}$$

where ${\hat{e}}_{i}=e\left(X_{i},\hat{\beta }\right)$ is the estimated IPS for subject i, Note that the normalization terms in ${\hat{\mu }}_{j}^{c}\left(IPW\right)$, $N^{-1}\sum_{i=1}^{N}\left(Z_{i}/{\hat{e}}_{i}\right)$ and $N^{-1}\sum_{i=1}^{N}\left\{\left(1-Z_{i}\right)/\left(1-{\hat{e}}_{i}\right)\right\}$, converge in probability to 1. The normalization terms are canceled out for ${\hat{\mu }}_{j}^{a}\left(IPW\right)$ and ${\hat{\mu }}_{j}^{n}\left(IPW\right)$.

In Theorem 2, we present the asymptotic properties of the proposed IPW estimators. Their asymptotic variances are presented in Appendix B.

**Theorem 2** *(Asymptotic distributions of the IPW estimators) Under Assumptions 1, 3 and 4, the random quantities* $n^{1/2}\left\{{\hat{\mu }}_{j}^{c}\left(IPW\right)-\mu_{j}^{c}\right\}$ , $n^{1/2}\left\{{\hat{\mu }}_{j}^{a}\left(IPW\right)-\mu_{j}^{a}\right\}$, *and* $n^{1/2}\left\{{\hat{\mu }}_{j}^{n}\left(IPW\right)-\mu_{j}^{n}\right\}$ *converge in distribution to mean-zero normal random variables*.

### Augmented IPW

Robins et al. [11] proposed an augmented IPW (AIPW) estimator for the average treatment effect (ATE). This estimator is consistent for the ATE when either the treatment or the outcome model is correctly specified. In addition, the AIPW estimator is semiparametrically efficient if both models are correctly specified. We adopt this AIPW estimator for profiling compliers and noncompliers to obtain potential accuracy and precision gains, when compared to the proposed IPW estimators.

To develop the AIPW estimators, we augment treatment models into the IPW estimators. Let *m*_1*i*_ and *m*_0*i*_ denote the mean models of *D* for subject *i* if that subject had been assigned to *Z* = 1 and *Z* = 0 groups:

$$\begin{array}{c} m_{1i}=E\left[D_{i}|Z_{i}=1,X_{i}\right],\;m_{0i}=E\left[D_{i}|Z_{i}=0,X_{i}\right]. \end{array}$$

Under Assumptions 3, *m*_1*i*_ = *E*[*D*_*i*_(1)|*X*_*i*_] and *m*_0*i*_ = *E*[*D*_*i*_(0)|*X*_*i*_]. We augment these treatment models into the IPW representations in Theorem 1, which gives the following theorem for the doubly robustness of the AIPW representations.

**Theorem 3** *(Doubly robustness of the AIPW representations) Under Assumptions 1, 3 and 4, the following AIPW representations correctly identify the means of X*_*ij*_ *for compliers, always-takers, and never-takers if either the IPS or treatment model is correctly specified*:

$$\begin{array}{ccc} \mu_{j}^{c} & = & \frac{E[\frac{Z_{i}D_{i}X_{ij}-\left(Z_{i}-e_{i}\right)m_{1i}X_{ij}}{e_{i}}]-E[\frac{\left(1-Z_{i}\right)D_{i}X_{ij}+\left(Z_{i}-e_{i}\right)m_{0i}X_{ij}}{1-e_{i}}]}{E[\frac{Z_{i}D_{i}-\left(Z_{i}-e_{i}\right)m_{1i}}{e_{i}}]-E[\frac{\left(1-Z_{i}\right)D_{i}+\left(Z_{i}-e_{i}\right)m_{0i}}{1-e_{i}}]},\\ \mu_{j}^{a} & = & \frac{E[\frac{\left(1-Z_{i}\right)D_{i}X_{ij}+\left(Z_{i}-e_{i}\right)m_{0i}X_{ij}}{1-e_{i}}]}{E[\frac{\left(1-Z_{i}\right)D_{i}+\left(Z_{i}-e_{i}\right)m_{0i}}{1-e_{i}}]},\\ \mu_{j}^{n} & = & \frac{E[\frac{Z_{i}\left(1-D_{i}\right)X_{ij}-\left(Z_{i}-e_{i}\right)\left(1-m_{1i}\right)X_{ij}}{e_{i}}]}{E[\frac{Z_{i}\left(1-D_{i}\right)-\left(Z_{i}-e_{i}\right)\left(1-m_{1i}\right)}{e_{i}}]}. \end{array}$$

The Proof for Theorem 3 is presented in Appendix C.

For estimation, we assume that *m*_1*i*_ and *m*_0*i*_ are parameterized with regression parameter vectors, *α*_1_ and *α*_0_, as follows:

$$\begin{array}{c} m_{1i}=m_{1}\left(X_{i},\alpha_{1}\right),\;m_{0i}=m_{0}\left(X_{i},\alpha_{0}\right). \end{array}$$

In practice, we use logistic regression models to estimate the treatment expectations: for *z* = 0, 1, $m_{z}\left(X_{i},\alpha_{z}\right)=\text{exp}\left(X_{i}^{T}\alpha_{z}\right)/\left\{1+\text{exp}\left(X_{i}^{T}\alpha_{z}\right)\right\}$. We denote by ${\hat{\alpha }}_{1}$ and ${\hat{\alpha }}_{0}$ the ML estimators for *α*_1_ and *α*_0_, respectively. These ML estimators are obtained by fitting the logistic regression models of *D* on *X* to the groups of *Z* = 1 and *Z* = 0, respectively. We denote by ${\hat{m}}_{1i}=m_{1}\left(X_{i},{\hat{\alpha }}_{1}\right)$ and ${\hat{m}}_{0i}=m_{0}\left(X_{i},{\hat{\alpha }}_{0}\right)$ the estimated treatment means. Based on Theorem 3, we suggest the following AIPW estimators:

$$\begin{array}{ccc} {\hat{\mu }}_{j}^{c}\left(AIPW\right) & = & \frac{\frac{\sum_{i=1}^{N}\left({\hat{m}}_{1i}-{\hat{m}}_{0i}\right)X_{ij}}{N}+\frac{\sum_{i=1}^{N}\frac{Z_{i}\left(D_{i}-{\hat{m}}_{1i}\right)X_{ij}}{{\hat{e}}_{i}}}{\sum_{i=1}^{N}\frac{Z_{i}}{{\hat{e}}_{i}}}-\frac{\sum_{i=1}^{N}\frac{\left(1-Z_{i}\right)\left(D_{i}-{\hat{m}}_{0i}\right)X_{ij}}{1-{\hat{e}}_{i}}}{\sum_{i=1}^{N}\frac{1-Z_{i}}{1-{\hat{e}}_{i}}}}{\frac{\sum_{i=1}^{N}\left({\hat{m}}_{1i}-{\hat{m}}_{0i}\right)}{N}+\frac{\sum_{i=1}^{N}\frac{Z_{i}\left(D_{i}-{\hat{m}}_{1i}\right)}{{\hat{e}}_{i}}}{\sum_{i=1}^{N}\frac{Z_{i}}{{\hat{e}}_{i}}}-\frac{\sum_{i=1}^{N}\frac{\left(1-Z_{i}\right)\left(D_{i}-{\hat{m}}_{0i}\right)}{1-{\hat{e}}_{i}}}{\sum_{i=1}^{N}\frac{1-Z_{i}}{1-{\hat{e}}_{i}}}},\\ {\hat{\mu }}_{j}^{a}\left(AIPW\right) & = & \frac{\frac{\sum_{i=1}^{N}{\hat{m}}_{0i}X_{ij}}{N}+(\sum_{i=1}^{N}\frac{1-Z_{i}}{1-{\hat{e}}_{i}}{)}^{-1}\sum_{i=1}^{N}\frac{\left(1-Z_{i}\right)\left(D_{i}-{\hat{m}}_{0i}\right)X_{ij}}{1-{\hat{e}}_{i}}}{\frac{\sum_{i=1}^{N}{\hat{m}}_{0i}}{N}+(\sum_{i=1}^{N}\frac{1-Z_{i}}{1-{\hat{e}}_{i}}{)}^{-1}\sum_{i=1}^{N}\frac{\left(1-Z_{i}\right)\left(D_{i}-{\hat{m}}_{0i}\right)}{1-{\hat{e}}_{i}}},\\ {\hat{\mu }}_{j}^{n}\left(AIPW\right) & = & \frac{\frac{\sum_{i=1}^{N}\left(1-{\hat{m}}_{1i}\right)X_{ij}}{N}-(\sum_{i=1}^{N}\frac{Z_{i}}{{\hat{e}}_{i}}{)}^{-1}\sum_{i=1}^{N}\frac{Z_{i}\left(D_{i}-{\hat{m}}_{1i}\right)X_{ij}}{{\hat{e}}_{i}}}{\frac{\sum_{i=1}^{N}\left(1-{\hat{m}}_{1i}\right)}{N}-(\sum_{i=1}^{N}\frac{Z_{i}}{{\hat{e}}_{i}}{)}^{-1}\sum_{i=1}^{N}\frac{Z_{i}\left(D_{i}-{\hat{m}}_{1i}\right)}{{\hat{e}}_{i}}}. \end{array}$$

In Theorem 4 below, we present the asymptotic properties of the AIPW estimators. Their asymptotic variances are presented in Appendix D.

**Theorem 4** *(Asymptotic distributions of the AIPW estimators) Under Assumptions 1, 3 and 4, the random quantities* $n^{1/2}\left\{{\hat{\mu }}_{j}^{c}\left(AIPW\right)-\mu_{j}^{c}\right\}$ , $n^{1/2}\left\{{\hat{\mu }}_{j}^{a}\left(AIPW\right)-\mu_{j}^{a}\right\}$, *and* $n^{1/2}\left\{{\hat{\mu }}_{j}^{n}\left(AIPW\right)-\mu_{j}^{n}\right\}$ *converge in distribution to mean-zero normal random variables*.

## Simulation

The proposed IPW and AIPW estimators were evaluated based on Monte Caro simulations with unconfounded and confounded IVs. Because the MH approach is valid for unconfounded IVs, we also included the MH estimators in the simulations with an unconfounded instrument. Two covariates, *X*_1_ and *X*_2_, were generated from a multivariate normal distribution with a zero mean vector and a compound symmetric covariance matrix with unit variance and a correlation coefficient of 0.5. Once these normal covariates were generated, *X*_2_ was dichotomized to be a binary variable: *I*(*X*_2_ > 0).

The instrument Z was generated from the following logistic regression function:

$$\begin{array}{c} e\left(X,\beta \right)=\frac{\text{exp}(\beta_{o}+\beta_{1}X_{1}+\beta_{2}X_{2})}{1+\text{exp}(\beta_{o}+\beta_{1}X_{1}+\beta_{2}X_{2})}. \end{array}$$

To simulate different degrees of confounding for the instrument Z, we used the following values for (*β*_1_, *β*_2_): (*β*_1_, *β*_2_) = (0, 0) for no confounding, (*β*_1_, *β*_2_) = (log 1.2, log 1.5) for mild confounding, and (*β*_1_, *β*_2_) = (log 1.5, log 2) for moderate confounding. The intercept term *β*_*o*_ was selected to have the marginal mean of *Z* be approximately equal to 0.30 for all three confounding scenarios for the instrument.

A compliance class *U* was defined such that *U* = 0, 1 and 2 indicated a never-taker, an always-taker, and a complier, respectively. The probability of being a complier was a logistic regression function:

$$\begin{array}{c} P\left[U=2|X\right]=\frac{\text{exp}(-0.5+0.3X_{1}-0.5X_{2})}{1+\text{exp}(-0.5+0.3X_{1}-0.5X_{2})}. \end{array}$$

The probabilities of a never-taker and an always-taker were the same as (1 − *P*[*U* = 2|*X*])/2. Then, *D* was a deterministic function of *Z* and *U*: *D* = *ZI*(*U* = 2) + *UI*(*U* ≠ 2). These probability models for compliers and noncompliers were used across all confounding scenarios for the instrument. Our simulation models yielded approximately *P*[*U* = 2] = 0.32 and *P*[*U* = 0] = *P*[*U* = 1] = 0.34.

By construction of our simulations, the true treatment model was not exactly a logistic function; hence there was a model misspecification for the treatment model because logistic models were used to estimate *m*_1*i*_ and *m*_0*i*_. However, our simulation results demonstrated that the AIPW estimators were robust to this mild model misspecification. The reason the true treatment model was not a logistic model is as follows. The stratum (*Z* = 1, *D* = 1) contains compliers and always takers, and therefore *m*_1*i*_ = *P*[*U*_*i*_ = 2|*X*_*i*_] + *P*[*U*_*i*_ = 1|*X*_*i*_]. The stratum (*Z* = 0, *D* = 1) contains only always takers, and hence *m*_0*i*_ = *P*[*U*_*i*_ = 1|*X*_*i*_]. Because *P*[*U* = 0|*X*] = *P*[*U* = 1|*X*] = (1 − *P*[*U* = 2|*X*])/2 under our simulation models, we have *m*_1*i*_ = (1 + *P*[*U*_*i*_ = 2|*X*_*i*_])/2 and *m*_0*i*_ = (1 − *P*[*U*_*i*_ = 2|*X*_*i*_])/2, which are not logistic functions.

We presented the performance of the profiling estimators for continuous *X*_1_ and dichotomous *X*_2_. The true means of *X*_1_ and *X*_2_ for compliers and noncompliers were calculated based on a very large sample size with several iterations. These true means are determined by the probability models for the compliance classes; hence they were the same for all confounding scenarios for the instrument. The true means of *X*_1_ for compliers, always-takers and never-takers were 0.133, -0.064 and -0.064, respectively. The true means of *X*_2_ for those compliance classes were 0.457, 0.520 and 0.520, respectively. The sample sizes were from 500 to 6000. The number of simulations was 500 for a given sample size.

To compare different methods, we calculated the bias, empirical standard error (ESE), and 95% confidence interval coverage rate (CR). These performance measures were calculated based on the 500 simulated data sets under each simulation scenario at a given sample size. Bias was obtained by taking the absolute value of the average of the 500 values of (estimate—true value). ESE was the empirical Monte Carlo standard error of the 500 estimates. The standard error estimates of the MH estimators were obtained by using the R package ivdesc [12]. Those of the IPW and AIPW estimators were obtained based on Theorems 2 and 4. CR was the empirical coverage of 95% confidence intervals using the estimated standard errors across the 500 simulated data sets.

**Fig 1:**
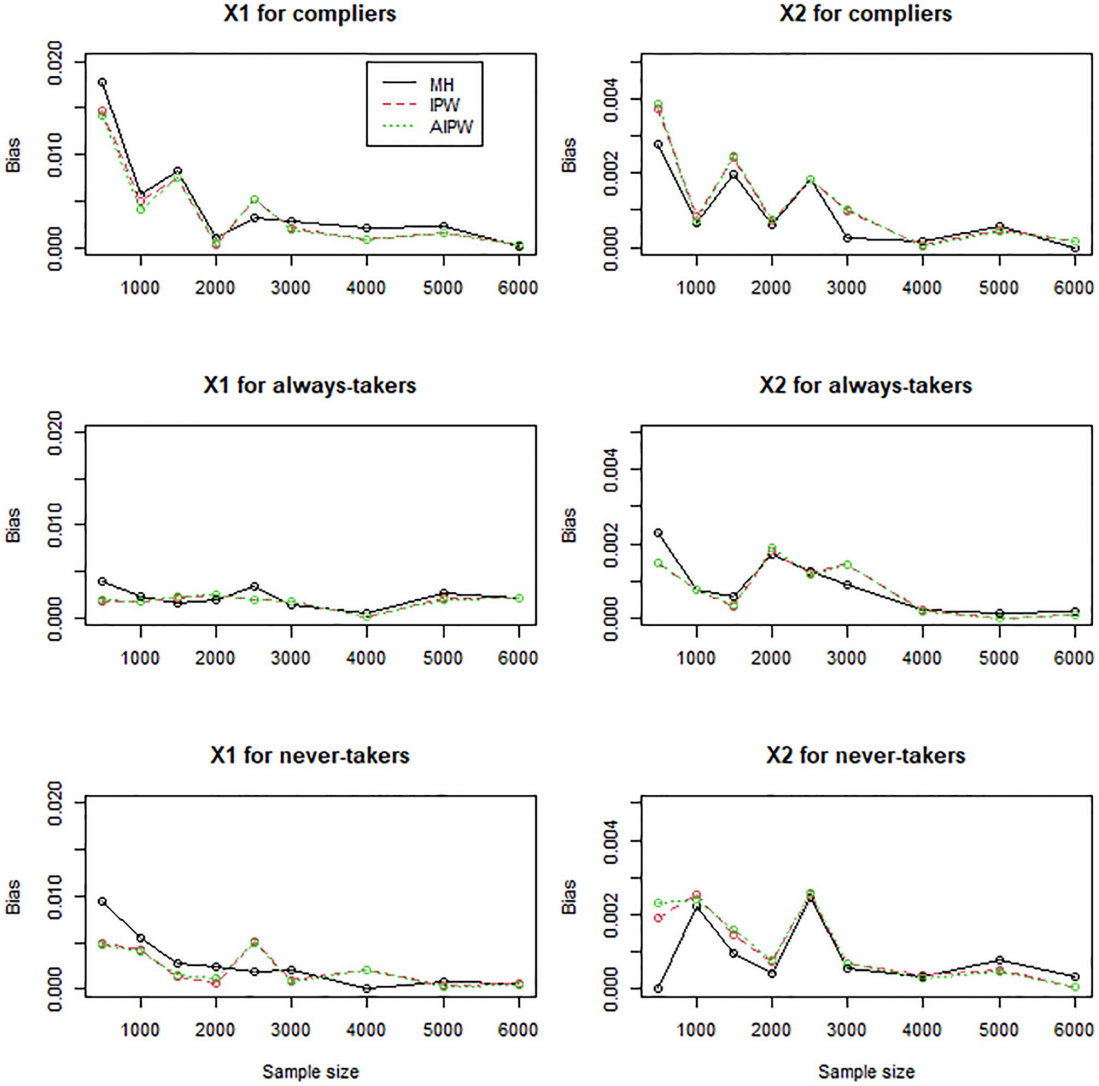
Bias of the MH, IPW, and AIPW estimators for continuous *X*_1_ and dichotomous *X*_2_ when the instrument is not confounded with the compliance behavior.

**Fig 2:**
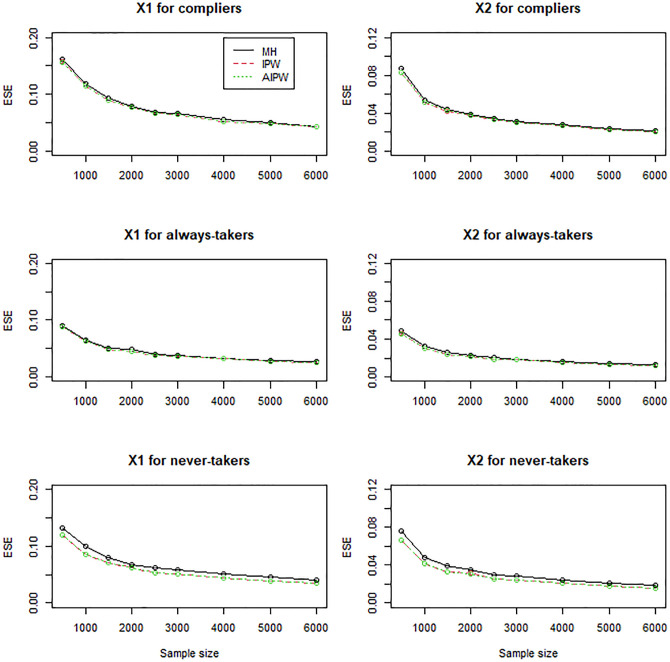
Empirical standard errors (ESE) of the MH, IPW, and AIPW estimators for continuous *X*_1_ and dichotomous *X*_2_ when the instrument is not confounded with the compliance behavior.

**Fig 3:**
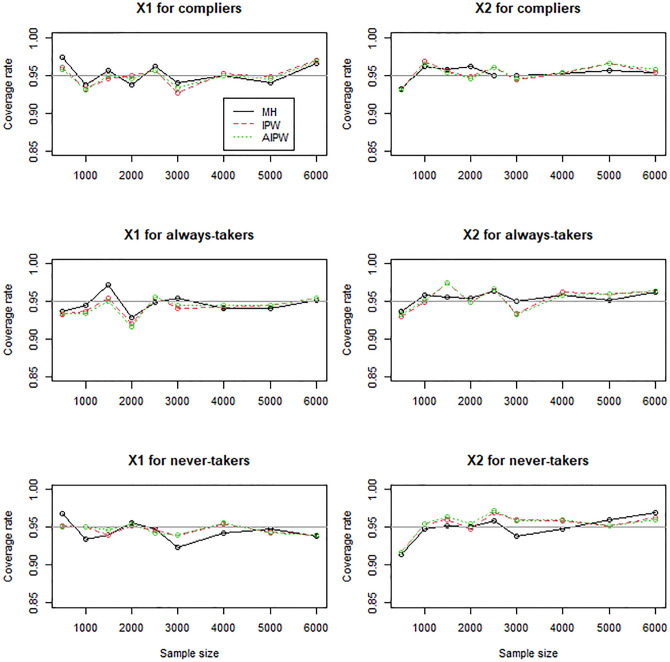
Coverage rates of the MH, IPW, and AIPW estimators for continuous *X*_1_ and dichotomous *X*_2_ when the instrument is not confounded with the compliance behavior.

In general, the IPW and AIPW estimators performed similarly to the MH estimators when the instrument was randomly assigned (Figs 1–3), except that the IPW and AIPW estimators of *X*_1_ and *X*_2_ for never-takers were slightly less variable than the corresponding MH estimators (Fig 2). All the methods yielded valid estimators to profile compliers and noncompliers (Figs 1–3): the bias reduced to zero, and the coverage rates converged to the target coverage rate of 95% as the sample size increased.

**Fig 4:**
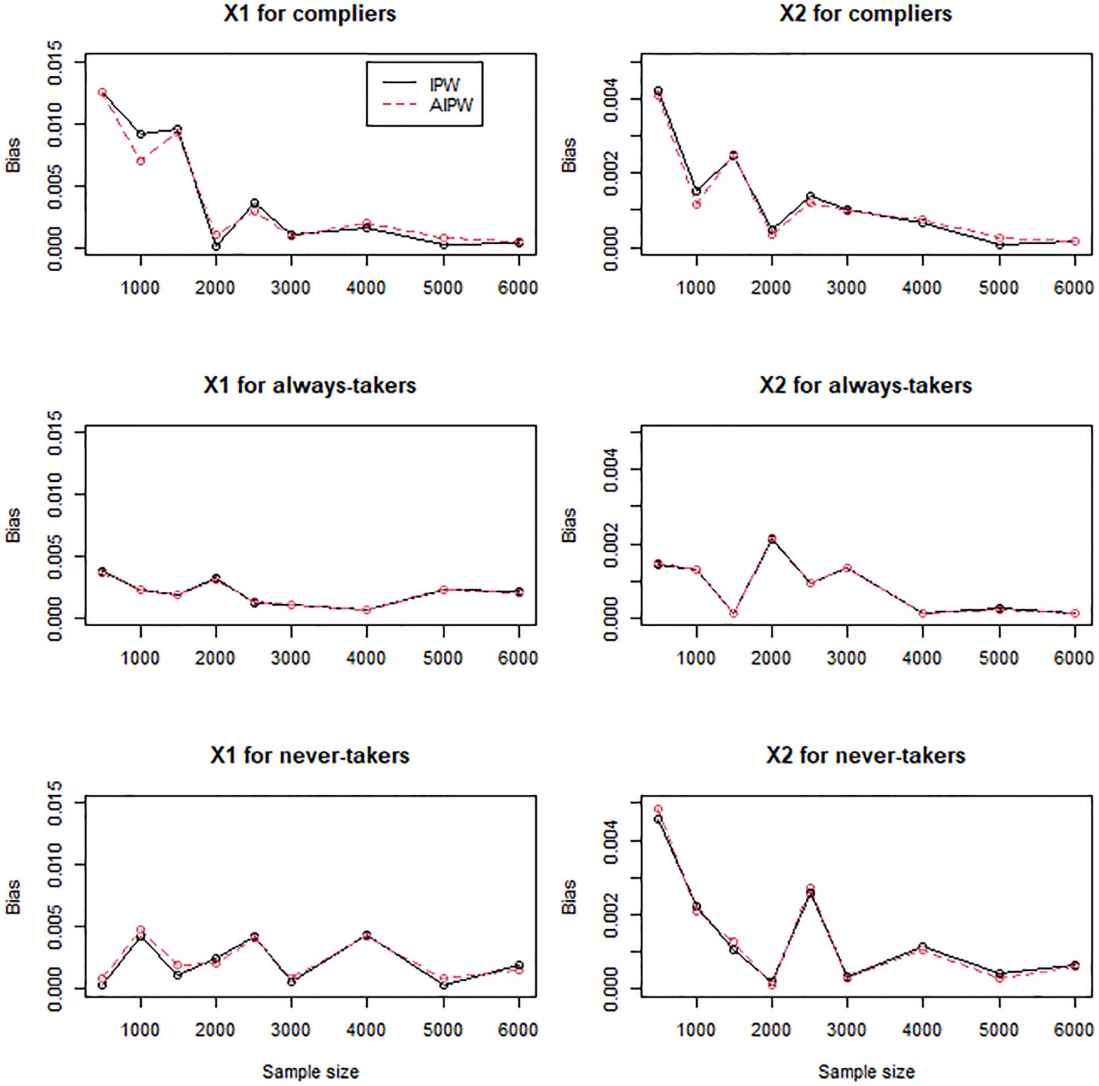
Bias of the IPW and AIPW estimators for continuous *X*_1_ and dichotomous *X*_2_ when the instrument is mildly confounded with the compliance behavior.

**Fig 5:**
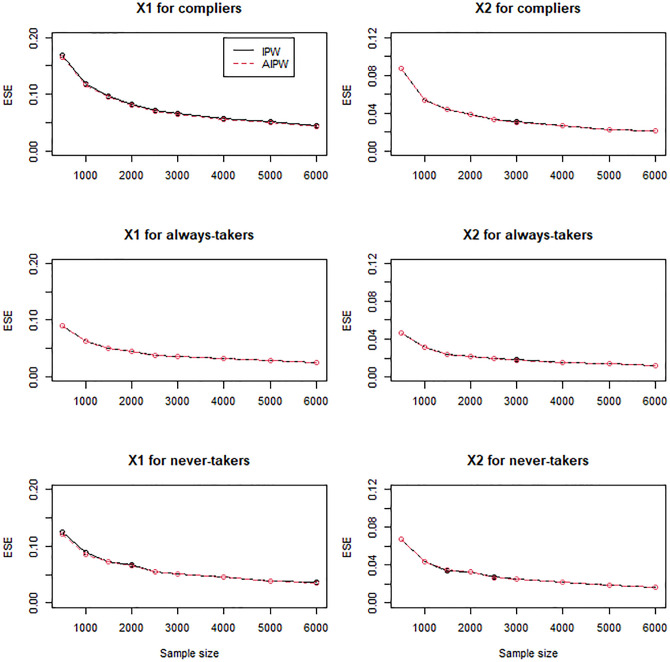
Empirical standard errors (ESE) of the IPW and AIPW estimators for continuous *X*_1_ and dichotomous *X*_2_ when the instrument is mildly confounded with the compliance behavior.

**Fig 6:**
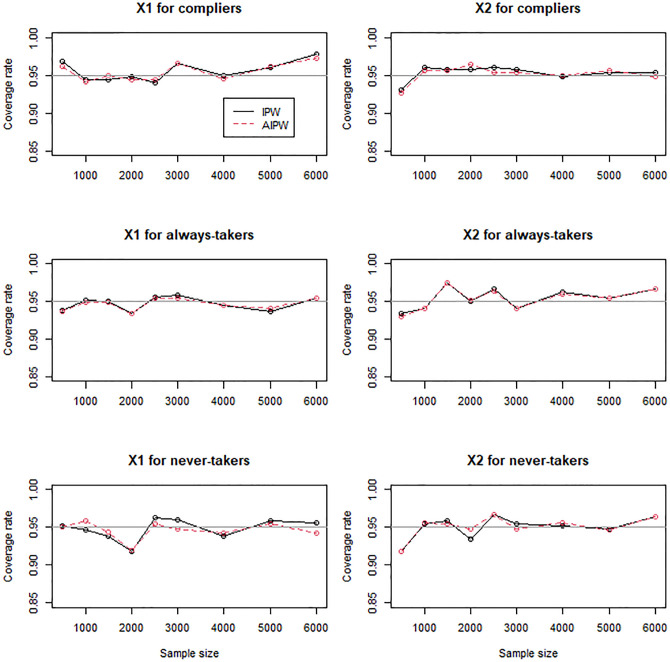
Coverage rates of the IPW and AIPW estimators for continuous *X*_1_ and dichotomous *X*_2_ when the instrument is mildly confounded with the compliance behavior.

**Fig 7:**
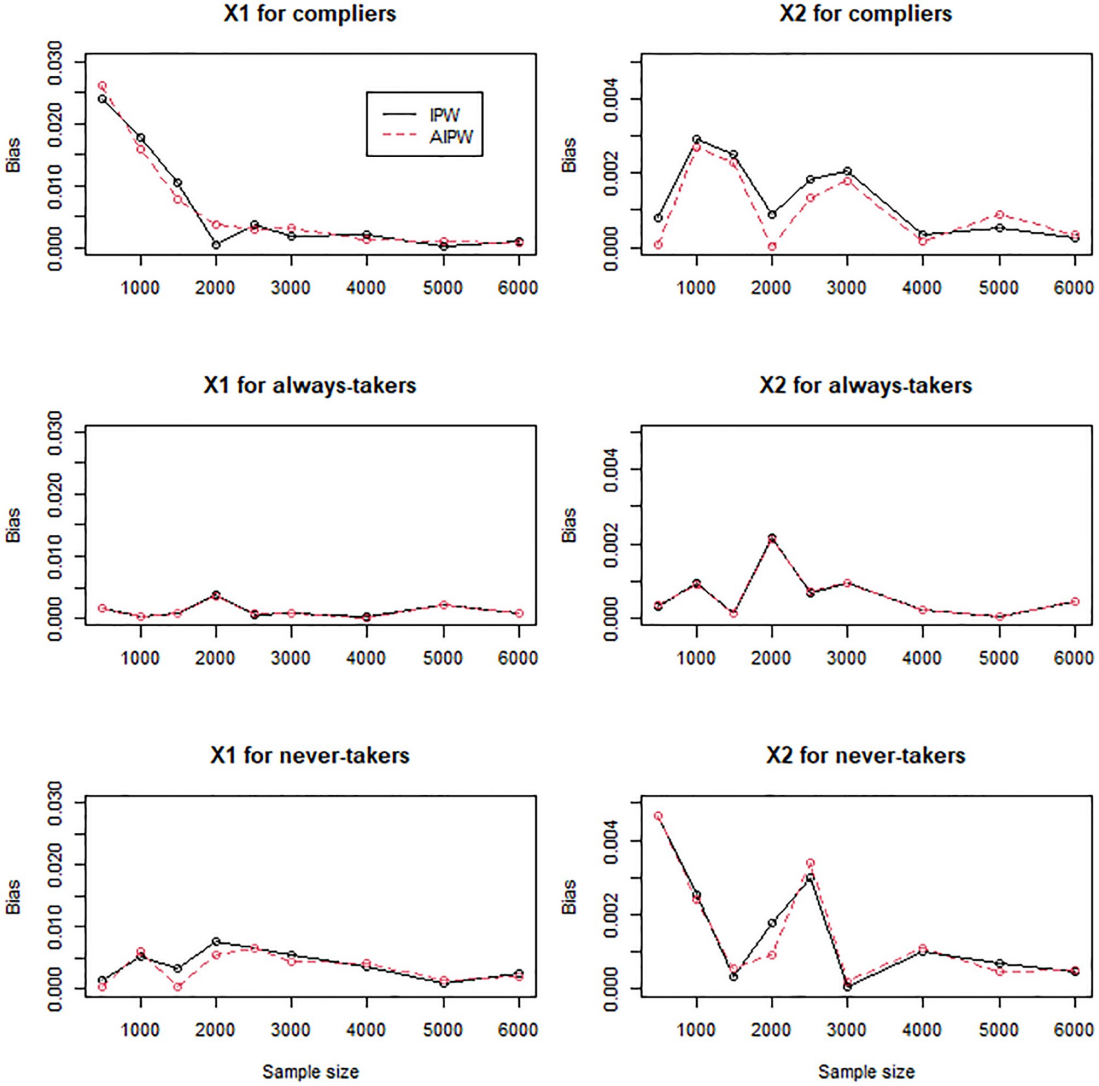
Bias of the IPW and AIPW estimators for continuous *X*_1_ and dichotomous *X*_2_ when the instrument is moderately confounded with the compliance behavior.

**Fig 8:**
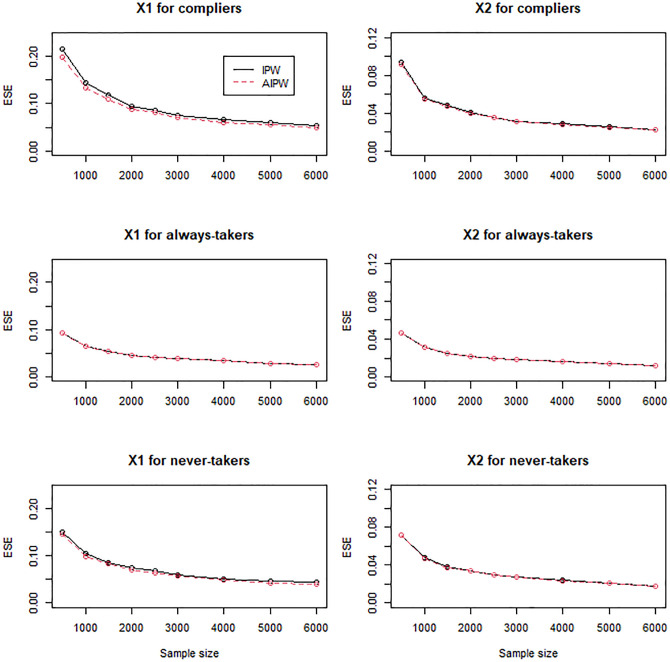
Empirical standard errors (ESE) of the IPW and AIPW estimators for continuous *X*_1_ and dichotomous *X*_2_ when the instrument is moderately confounded with the compliance behavior.

**Fig 9:**
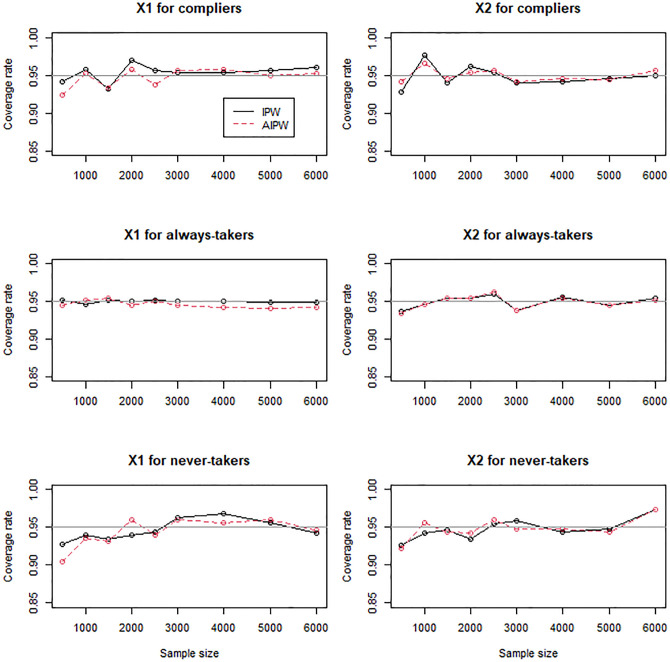
Coverage rates of the IPW and AIPW estimators for continuous *X*_1_ and dichotomous *X*_2_ when the instrument is moderately confounded with the compliance behavior.

For the scenarios with mildly (Figs 4–6) and moderately (Figs 7–9) confounded IVs, we compared only the IPW and AIPW estimators because the MH estimators were not valid in such settings. The bias of the IPW and AIPW estimators reduced to zero (Figs 4 and 7), and the coverage rates of those converged to the target coverage rate of 95% as the sample size increased (Figs 6 and 9), which implied that the proposed point and standard error estimates addressed the confounded instruments appropriately. The AIPW method yielded coverage rates that were slightly closer to the target rate than the IPW method in estimating the means of *X*_1_ and *X*_2_ for never-takers when the instrument was mildly confounded (Fig 6). In addition, the AIPW estimator was slightly less variable than the IPW estimator in estimating the mean of *X*_1_ for compliers when the instrument was moderately confounded (Fig 8). Except those cases, in general, the IPW and AIPW estimators performed similarly.

## Application

We applied the proposed methodology to the data for the causal effects of education on earnings. The data set included 3010 observations between 14 and 24 years in 1996 from the National Longitudinal Survey of Young Men (NLSYM), originally analyzed by Card [4], and afterward by several statistical articles [9, 13, 14]. In his analysis, the outcome was log-transformed wages in the survey year (1976), the treatment was years of education, and the IV was a binary indicator for growing up near a 4-year college in 1966. In our analysis, instead of years of education, we used education beyond high school (EBH) as the treatment, as in Tan [9]. In other words, the treatment variable *D* was an indicator for whether the years of education were more than 12 years. Our outcome Y was log wages in cents per hour in 1976. The instrument was proximity to a 4-year college in 1966. The compliers in our analysis were those who would have attained EBH if they had lived close to a 4-year college. There were 68.2% who grew up near a 4-year college and 50.5 had EBH. The covariates X to adjust for confounding included age, age squared, race, an indicator for living with both mother and father at age 14, an indicator for living with single mom at age 14, an indicator for living with stepparents at age 14, and residence in the South in 1966.

We first estimated the LATE of EBH on log wages without adjusting for any covariates. Based on Angrist et al. [1], this LATE was the intent-to-treat effect of the IV on log wages divided by that of the IV on EBH. Using the R package ivmodel [15], the LATE was calculated as 1.28 with a 95% confidence interval of (0.84, 1.72). Next, we estimated the LATE of EBH with covariates *X* using the approach of Abadie [3], which uses the kappa weights based on the IPS of college proximity. We fitted a logistic regression model of college proximity on the covariates X to estimate the IPS. The estimated IPS model revealed that residence in the South was strongly associated with the IV with an odds ratio of 0.38. Using the R package LARF [16], the calculated LATE was 0.87 with a 95% confidence interval of (0.49, 1.26). Therefore, both LATE estimates indicated that EBH increased future wages.

**Fig 10:**
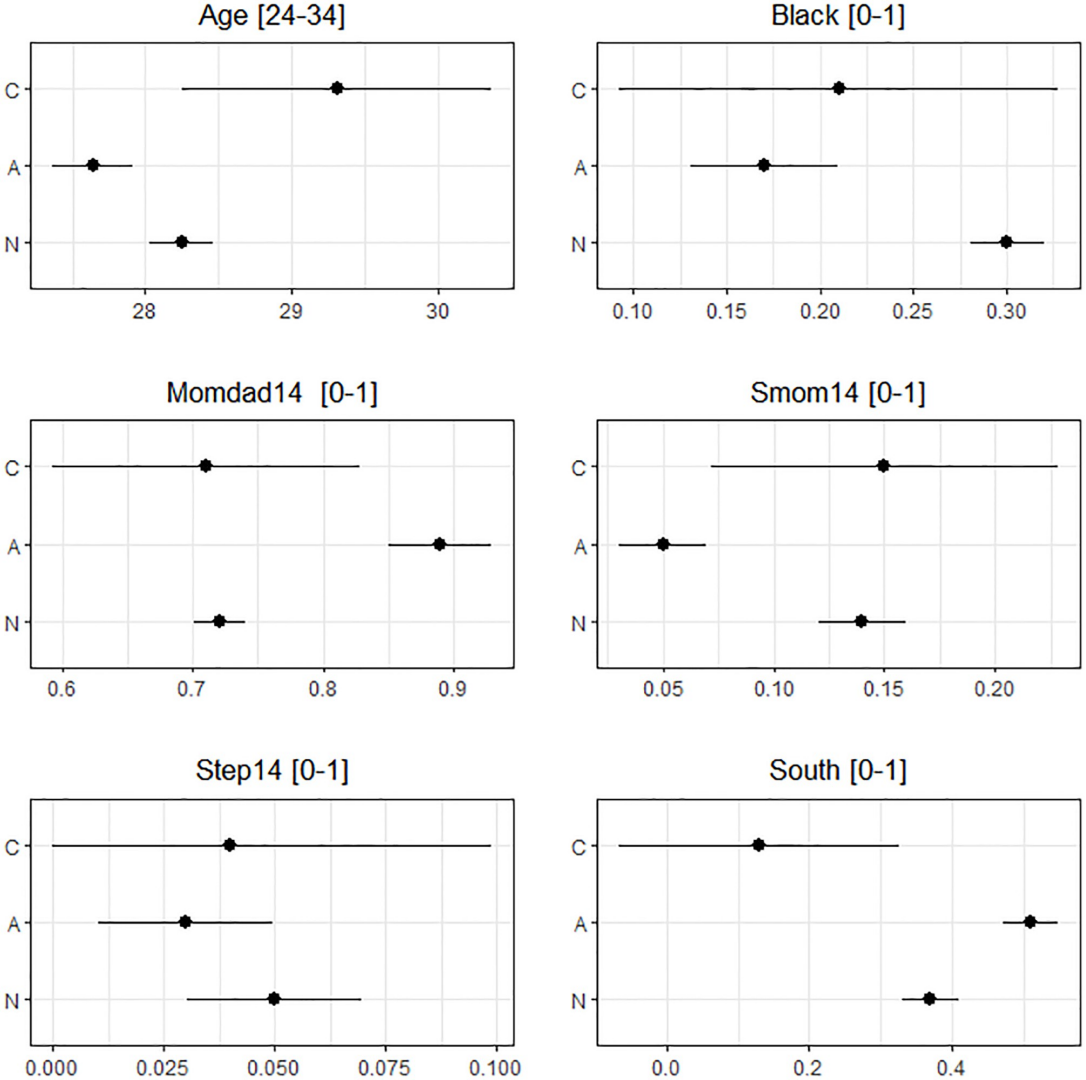
MH estimates of the means and their 95% confidence intervals for the characteristics of the complier and noncomplier subpopulations in the NLSYM data. C, A, and N on the Y-axis indicate the subpopulations of compliers, always-takers, and never-takers, respectively. Momdad14 is an indicator for living with both mother and father at age 14. Smom14 is an indicator for living with single mom at age 14. Step14 is an indicator for living with stepparents at age 14. South is an indicator for residence in the South in 1966.

**Fig 11:**
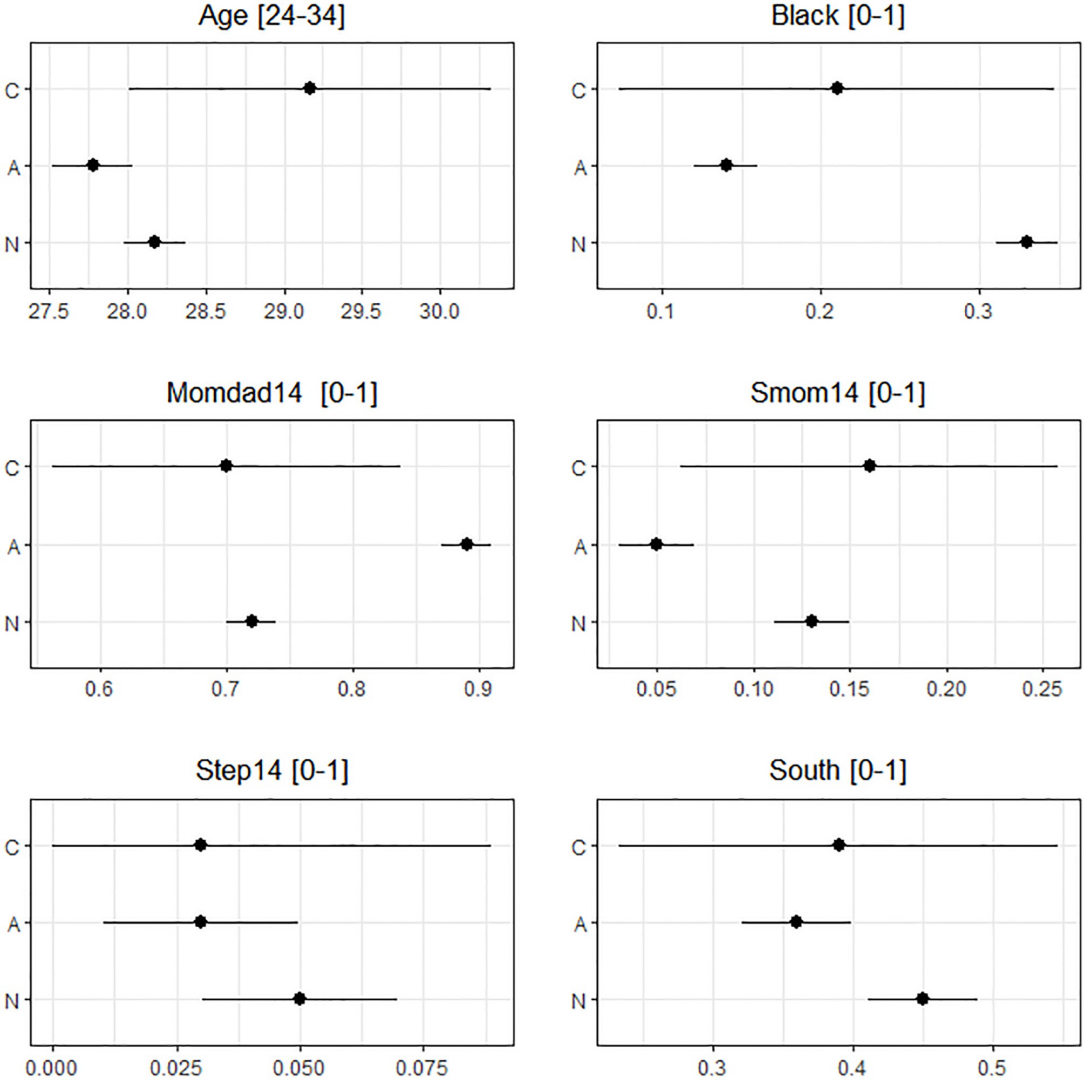
AIPW estimates of the means and their 95% confidence intervals for the characteristics of the complier and noncomplier subpopulations in the NLSYM data. C, A, and N on the Y-axis indicate the subpopulations of compliers, always-takers, and never-takers, respectively. Momdad14 is an indicator for living with both mother and father at age 14. Smom14 is an indicator for living with single mom at age 14. Step14 is an indicator for living with stepparents at age 14. South is an indicator for residence in the South in 1966.

To interpret these LATE estimates of EBH, it would be reasonable to characterize the compliers and noncompliers because they can be different in important ways, and the LATE addresses the causal effect only for the compliers. To this end, we used the MH approach and our weighing approach to estimate the covariate means of the compliers and noncompliers. Different profiling results were observed between the MH and our approaches for some covariates. The IPW and AIPW results were very similar, and thus we displayed only the MH (Fig 10) and AIPW (Fig 11) estimates and the corresponding 95% confidence intervals. Because of the strong correlation between residence in the South and the IV, the MH and AIPW methods gave very different profiling results for that covariate: the MH estimates implied that the compliers were significantly less likely to live in the South in 1996 than the noncompliers, but the compliers appeared to live in the South with a similar proportion to those of the noncompliers based on the AIPW estimates. Even though the MH and weighting approaches gave different results for some covariates, both approaches demonstrated that the compliers and noncompliers were different. The compliers were older than the noncompliers, less likely to be black than the never-takers, and more likely to live with single mother at age 14 than the always-takers.

## Conclusion

In this study, we proposed weighting estimators to profile compliers and noncompliers when the instrument is confounded with the compliance behavior. The proposed estimators are based on IPW and AIPW, which are frequently used for estimation of causal effects. The proposed estimators are also valid for instruments that are randomly assigned, and hence are more generally applicable than the MH estimators. The simulation study demonstrated that the proposed methods correctly account for confounded instruments. It also showed that the use of the proposed estimators can bring some precision gains in estimating the covariate means of noncompliers in randomized experiments when compared to the MH estimators. We applied our profiling approach to the data set from Card [4], where the compliers were those who would have attained EBH if they had lived near a 4-year college. Because residence in the South was strongly associated with EBH, the MH and our weighting approaches gave significantly different results for that covariate, which implies that accounting for significant correlations between the covariates and the IV is important to profile the compliers and noncompliers accurately.

Among the two proposed methods, the AIPW method yielded slightly better coverage rates and smaller standard errors than the IPW method for some simulation scenarios. In the analysis of the NLSYM data, the two methods gave almost identical estimates and standard errors for the baseline mean characteristics of compliers and noncompliers. Based on these observations, the AIPW method would be preferable to the IPW method, unless the treatment models for the AIPW are not severely misspecified.

We used ML to estimate the IPSs for the proposed IPW and AIPW estimators. This allowed us to derive their asymptotic distributions based on standard asymptotic theory. Based on the score equation, ML finds the parameter values such that the average of prediction errors is minimized. However, estimation of the IPS does not necessarily rely on ML. Because the PS is a balancing score [17], we can adopt estimation methods based on balancing covariates. One of those is the covariate balancing propensity score (CBPS) by Imai and Ratkovic [18], which estimates a parametric PS model by solving covariate balancing moment conditions. Adopting the CBPS into the IPS estimation involves estimating the regression parameters of the parametric IPS model by solving the covariate balancing moment conditions. Simulation studies demonstrated that CBPS can yield less biased and more stable PS weighting estimators than ML when either model misspecification or limited overlap exists for the PS [19–21]. Based on these simulation results, adopting CBPS would improve the robustness of the proposed weighting estimators.

A recent study demonstrated that adopting machine learning techniques such as lasso [22] and super learner [23] for outcome regression enhances the robustness of the AIPW estimator for the ATE to model misspecification by reducing bias and standard error [24]. To further improve the performance of the AIPW profiling estimators, we can adopt these machine learning techniques to estimate the treatment models. However, the development of the associated inferential procedures is complicated and merits further investigation.

## Appendix A: Proof of Theorem 1

The always-taker mean of *X*_*ij*_ is $\mu_{j}^{a}=\int xf_{j}^{a}\left(x\right)dx$. Based on Bayes’ theorem,

$$\begin{array}{c} f_{j}^{a}\left(x\right)=P\left[D_{i}\left(1\right)=D_{i}\left(0\right)=1|X_{ij}=x\right]f_{j}\left(x\right)/P\left[D_{i}\left(1\right)=D_{i}\left(0\right)=1\right]. \end{array}$$

Therefore, $\mu_{j}^{a}$ can be written as

$$\begin{array}{c} \mu_{j}^{a}=\frac{\int xP\left[D_{i}\left(1\right)=D_{i}\left(0\right)=1|X_{ij}=x\right]f_{j}\left(x\right)dx}{P[D_{i}\left(1\right)=D_{i}\left(0\right)=1]}. \end{array}$$ (5)

Under monotonicity, *D*_*i*_(0) = 1 implies *D*_*i*_(1) = 1. This leads to

$$\begin{array}{c} P\left[D_{i}\left(1\right)=D_{i}\left(0\right)=1|X_{ij}=x\right]=P\left[D_{i}\left(0\right)=1|X_{ij}=x\right]=E\left[D_{i}\left(0\right)|X_{ij}=x\right], \end{array}$$ (6)

and *P*[*D*_*i*_(1) = *D*_*i*_(0) = 1] = *E*[*D*_*i*_(0)]. As a result, the denominator of Eq (5) can be identified by *E*[(1 − *Z*_*i*_)*D*_*i*_/(1 − *e*_*i*_)]. Based on Eq (6), the numerator of Eq (5) can be written as

$$\begin{array}{c} \int E\left[xD_{i}\left(0\right)|X_{ij}=x\right]f_{j}\left(x\right)dx=E\left[X_{ij}D_{i}\left(0\right)\right], \end{array}$$

which is identified by Eq (4).

The never-taker mean of *X*_*ij*_ is $\mu_{j}^{n}=\int xf_{j}^{n}\left(x\right)dx$. Based on Bayes’ theorem,

$$\begin{array}{c} f_{j}^{n}\left(x\right)=P\left[D_{i}\left(1\right)=D_{i}\left(0\right)=0|X_{ij}=x\right]f_{j}\left(x\right)/P\left[D_{i}\left(1\right)=D_{i}\left(0\right)=0\right]. \end{array}$$

Therefore, $\mu_{j}^{n}$ can be written as

$$\begin{array}{c} \mu_{j}^{n}=\frac{\int xP\left[D_{i}\left(1\right)=D_{i}\left(0\right)=0|X_{ij}=x\right]f_{j}\left(x\right)dx}{P[D_{i}\left(1\right)=D_{i}\left(0\right)=0]}. \end{array}$$ (7)

Under monotonicity, *D*_*i*_(1) = 0 implies *D*_*i*_(0) = 0. This leads to

$$\begin{array}{c} P\left[D_{i}\left(1\right)=D_{i}\left(0\right)=0|X_{ij}=x\right]=P\left[D_{i}\left(1\right)=0|X_{ij}=x\right]=E\left[1-D_{i}\left(1\right)|X_{ij}=x\right], \end{array}$$ (8)

and *P*[*D*_*i*_(1) = *D*_*i*_(0) = 0] = *E*[1 − *D*_*i*_(1)]. Because *E*[*Z*_*i*_/*e*_*i*_] = 1, we can identify the denominator of (7) by

$$\begin{array}{c} E\left[1-D_{i}\left(1\right)\right]=E[\frac{Z_{i}}{e_{i}}-\frac{Z_{i}D_{i}}{e_{i}}]=E[\frac{\left(1-Z_{i}\right)D_{i}}{e_{i}}]. \end{array}$$

Based on Eq (8), the numerator of Eq (7) can be written as

$$\begin{array}{c} \int E\left[x\left\{1-D_{i}\left(1\right)\right\}|X_{ij}=x\right]f_{j}\left(x\right)dx=E\left[X_{ij}\left\{1-D_{i}\left(1\right)\right\}\right]. \end{array}$$ (9)

It is worthwhile to note that

$$\begin{array}{c} E[\frac{Z_{i}X_{ij}}{e_{i}}]=E[E[\frac{Z_{i}X_{ij}}{e_{i}}|X_{i}]]=E[\frac{X_{ij}}{e_{i}}E\left[Z_{i}|X_{i}\right]]=E\left[X_{ij}\right]. \end{array}$$

Then, based on Eq (3), Eq (9) can be identified by

$$\begin{array}{c} E\left[X_{ij}\left\{1-D_{i}\left(1\right)\right\}\right]=E[\frac{Z_{i}X_{ij}\left(1-D_{i}\right)}{e_{i}}]. \end{array}$$

## Appendix B: Proof of Theorem 2

We can express the IPW estimator for $\mu_{j}^{c}$ as ${\hat{\mu }}_{j}^{c}\left(IPW\right)=\left(\mu_{1}-\mu_{2}\right)/\left(\mu_{3}-\mu_{4}\right)$, where

$$\begin{array}{ccc} \mu_{1} & = & (\sum_{i=1}^{N}\frac{Z_{i}}{{\hat{e}}_{i}}{)}^{-1}\sum_{i=1}^{N}\frac{Z_{i}D_{i}X_{ij}}{{\hat{e}}_{i}},\;\mu_{2}=(\sum_{i=1}^{N}\frac{1-Z_{i}}{1-{\hat{e}}_{i}}{)}^{-1}\sum_{i=1}^{N}\frac{\left(1-Z_{i}\right)D_{i}X_{ij}}{1-{\hat{e}}_{i}},\\ \mu_{3} & = & (\sum_{i=1}^{N}\frac{Z_{i}}{{\hat{e}}_{i}}{)}^{-1}\sum_{i=1}^{N}\frac{Z_{i}D_{i}}{{\hat{e}}_{i}},\;\mu_{4}=(\sum_{i=1}^{N}\frac{1-Z_{i}}{1-{\hat{e}}_{i}}{)}^{-1}\sum_{i=1}^{N}\frac{\left(1-Z_{i}\right)D_{i}}{1-{\hat{e}}_{i}}. \end{array}$$

Then, the estimator of $\theta =\{\mu_{1},\mu_{2},\mu_{3},\mu_{4},\beta^{T},\mu_{j}^{c}\left(IPW\right){\}}^{T}$ can be viewed as a solution to the following estimating equations:

$$\begin{array}{c} 0=\sum_{i=1}^{N}\phi_{i}\left(\theta \right)=\sum_{i=1}^{N}[\begin{array}{c} \left\{Z_{i}/e_{i}\left(\beta \right)\right\}\left(D_{i}X_{ij}-\mu_{1}\right)\\ \left\{\left(1-Z_{i}\right)/\left(1-e_{i}\left(\beta \right)\right)\right\}\left(D_{i}X_{ij}-\mu_{2}\right)\\ \left\{Z_{i}/e_{i}\left(\beta \right)\right\}\left(D_{i}-\mu_{3}\right)\\ \left\{\left(1-Z_{i}\right)/\left(1-e_{i}\left(\beta \right)\right)\right\}\left(D_{i}-\mu_{4}\right)\\ S_{\beta }\left(X_{i},\beta \right)\\ \left(\mu_{1}-\mu_{2}\right)/\left(\mu_{3}-\mu_{4}\right)-\mu_{j}^{c}\left(IPW\right) \end{array}], \end{array}$$

where *S*_*β*_(*X*_*i*_, *β*) is the likelihood score equation for the IPS model. The last element of $\hat{\theta }$, obtained as a solution to $0=\sum_{i=1}^{N}\phi_{i}\left(\theta \right)$, is ${\hat{\mu }}_{j}^{c}\left(IPW\right)$. Therefore, ${\hat{\mu }}_{j}^{c}\left(IPW\right)$ is an M-estimator, which has an asymptotic normal distribution [25]. The variance of $\hat{\theta }$ is estimated by *N*^−1^*A*^−1^*BA*^−*T*^, with $A=-N^{-1}\sum_{i=1}^{N}\partial \phi_{i}{\left(\theta \right)/\partial \theta |}_{\theta =\hat{\theta }}$ and $B=N^{-1}\sum_{i=1}^{N}\phi_{i}\left(\theta \right)\phi_{i}{\left(\theta \right)}^{T}{|}_{\theta =\hat{\theta }}$. The estimated variance of ${\hat{\mu }}_{j}^{c}\left(IPW\right)$ is the last diagonal element of *N*^−1^*A*^−1^*BA*^−*T*^. The asymptotic normal distributions of ${\hat{\mu }}_{j}^{a}\left(IPW\right)$ and ${\hat{\mu }}_{j}^{n}\left(IPW\right)$ can be demonstrated in a similar way.

## Appendix C: Proof of Theorem 3

We demonstrate that the AIPW representation of $\mu_{j}^{c}$ is doubly robust. The doubly robustness of the AIPW representations of $\mu_{j}^{a}$ and $\mu_{j}^{n}$ can be shown in a similar way, and hence the proofs of those are omitted. The AIPW representation for the denominator of $\mu_{j}^{c}$ in Theorem 3 is known to be doubly robust for *E*[*D*_*i*_(1) − *D*_*i*_(0)] [10, 26, 27], which means that it is equal to *E*[*D*_*i*_(1) − *D*_*i*_(0)] if either the IPS or treatment model is correctly specified. Therefore, it is sufficient to show that the AIPW representation for the numerator of $\mu_{j}^{c}$ in Theorem 3 is doubly robust for *E*[*X*_*ij*_*D*(1)] − *E*[*X*_*ij*_*D*(0)].

We can express the first term in the numerator of $\mu_{j}^{c}$ in Theorem 3 as

$$\begin{array}{c} E[\frac{Z_{i}D_{i}X_{ij}-\left(Z_{i}-e_{i}\right)m_{1i}X_{ij}}{e_{i}}]=E[\frac{Z_{i}D_{i}X_{ij}}{e_{i}}]-E[\frac{\left(Z_{i}-e_{i}\right)m_{1i}X_{ij}}{e_{i}}]. \end{array}$$ (10)

If the IPS is correctly specified, then [(*Z*_*i*_ − *e*_*i*_)*X*_*ij*_*m*_1*i*_/*e*_*i*_] = 0, and hence the above equation becomes *E*[*X*_*ij*_*D*(1)]. In addition, we can express Eq (10) as

$$\begin{array}{c} E[\frac{Z_{i}D_{i}X_{ij}-\left(Z_{i}-e_{i}\right)m_{1i}X_{ij}}{e_{i}}]=E[\frac{Z_{i}X_{ij}\left(D_{i}-m_{1i}\right)}{e_{i}}]-E\left[X_{ij}m_{1i}\right]. \end{array}$$ (11)

If *m*_1*i*_ is correctly specified, then *E*[*Z*_*i*_(*D*_*i*_ − *m*_1*i*_)*X*_*ij*_/*e*_*i*_] = 0 and *E*[*X*_*ij*_*m*_1*i*_] = *E*[*E*[*X*_*ij*_*D*_*i*_(1)|*X*_*i*_]] = *E*[*X*_*ij*_*D*_*i*_(1)]; hence Eq (11) becomes *E*[*X*_*i*_*jD*_*i*_(1)].

In a similar way, we can demonstrate that the second term in the numerator of $\mu_{j}^{c}$ in Theorem 3,

$$\begin{array}{c} E[\frac{\left(1-Z_{i}\right)D_{i}X_{ij}+\left(Z_{i}-e_{i}\right)m_{0i}X_{ij}}{1-e_{i}}], \end{array}$$ (12)

is doubly robust for *E*[*X*_*ij*_*D*_*i*_(0)]. Eq (12) can be expressed as

$$\begin{array}{c} E[\frac{\left(1-Z_{i}\right)D_{i}X_{ij}+\left(Z_{i}-e_{i}\right)m_{0i}X_{ij}}{1-e_{i}}]=E[\frac{\left(1-Z_{i}\right)D_{i}X_{ij}}{1-e_{i}}]+E[\frac{\left(Z_{i}-e_{i}\right)m_{0i}X_{ij}}{1-e_{i}}]. \end{array}$$ (13)

If the IPS is correctly specified, then *E*[(*Z*_*i*_ − *e*_*i*_)*X*_*ij*_*m*_0*i*_/(1 − *e*_*i*_)] = 0, and hence Eq (13) becomes *E*[*X*_*ij*_*D*_*i*_(0)]. In addition, we can express Eq (12) as

$$\begin{array}{c} E[\frac{\left(1-Z_{i}\right)D_{i}X_{ij}+\left(Z_{i}-e_{i}\right)m_{0i}X_{ij}}{1-e_{i}}]=E[\frac{\left(1-Z_{i}\right)X_{ij}\left(D_{i}-m_{0i}\right)}{1-e_{i}}]+E\left[X_{ij}m_{0i}\right]. \end{array}$$ (14)

If *m*_0*i*_ is correctly specified, then *E*[(1 − *Z*_*i*_)*X*_*ij*_(*D*_*i*_ − *m*_0*i*_)/{1 − *e*_*i*_}] = 0 and *E*[*X*_*ij*_*m*_0*i*_] = *E*[*E*[*X*_*ij*_*D*_*i*_(0)|*X*_*i*_]] = *E*[*X*_*ij*_*D*_*i*_(0)]; hence, Eq (14) becomes *E*[*X*_*ij*_*D*_*i*_(0)]. This completes the proof.

## Appendix D: Proof of Theorem 4

We can express the AIPW estimator for $\mu_{j}^{c}$ as ${\hat{\mu }}_{j}^{c}\left(AIPW\right)=\left(\eta_{1}+\eta_{2}-\eta_{3}\right)/\left(\eta_{4}+\eta_{5}-\eta_{6}\right)$, where

$$\begin{array}{ccc} \eta_{1} & = & \frac{\sum_{i=1}^{N}\left({\hat{m}}_{1i}-{\hat{m}}_{0i}\right)X_{ij}}{N},\;\eta_{2}=\frac{\sum_{i=1}^{N}\frac{Z_{i}\left(D_{i}-{\hat{m}}_{1i}\right)X_{ij}}{{\hat{e}}_{i}}}{\sum_{i=1}^{N}\frac{Z_{i}}{{\hat{e}}_{i}}},\;\eta_{3}=\frac{\sum_{i=1}^{N}\frac{\left(1-Z_{i}\right)\left(D_{i}-{\hat{m}}_{0i}\right)X_{ij}}{{\hat{e}}_{i}}}{\sum_{i=1}^{N}\frac{1-Z_{i}}{1-{\hat{e}}_{i}}}\\ \eta_{4} & = & \frac{\sum_{i=1}^{N}\left({\hat{m}}_{1i}-{\hat{m}}_{0i}\right)}{N},\;\eta_{5}=\frac{\sum_{i=1}^{N}\frac{Z_{i}\left(D_{i}-{\hat{m}}_{1i}\right)}{{\hat{e}}_{i}}}{\sum_{i=1}^{N}\frac{Z_{i}}{{\hat{e}}_{i}}},\;\eta_{6}=\frac{\sum_{i=1}^{N}\frac{\left(1-Z_{i}\right)\left(D_{i}-{\hat{m}}_{0i}\right)}{{\hat{e}}_{i}}}{\sum_{i=1}^{N}\frac{1-Z_{i}}{1-{\hat{e}}_{i}}} \end{array}$$

Then, the estimator of $\nu =\{\eta_{1},\eta_{2},\eta_{3},\eta_{4},\eta_{5},\eta_{6},\alpha_{1}^{T},\alpha_{0}^{T},\beta^{T},\mu_{j}^{c}\left(AIPW\right){\}}^{T}$ can be viewed as a solution to the following estimating equations:

$$\begin{array}{c} 0=\sum_{i=1}^{N}\psi_{i}\left(\theta \right)=\sum_{i=1}^{N}[\begin{array}{c} \left\{m_{1i}\left(\alpha_{1}\right)-m_{0i}\left(\alpha_{0}\right)\right\}X_{ij}-\eta_{1}\\ \left\{Z_{i}/e_{i}\left(\beta \right)\right\}\left[\left\{D_{i}-m_{1i}\left(\alpha_{1}\right)\right\}X_{ij}-\eta_{2}\right]\\ \left\{\left(1-Z_{i}\right)/\left(1-e_{i}\left(\beta \right)\right)\right\}\left[\left\{D_{i}-m_{0i}\left(\alpha_{0}\right)\right\}X_{ij}-\eta_{3}\right]\\ \left\{m_{1i}\left(\alpha_{1}\right)-m_{0i}\left(\alpha_{0}\right)\right\}-\eta_{4}\\ \left\{Z_{i}/e_{i}\left(\beta \right)\right\}\left\{D_{i}-m_{1i}\left(\alpha_{1}\right)-\eta_{5}\right\}\\ \left\{\left(1-Z_{i}\right)/\left(1-e_{i}\left(\beta \right)\right)\right\}\left\{D_{i}-m_{0i}\left(\alpha_{0}\right)-\eta_{6}\right\}\\ Z_{i}S_{1}\left(D_{i},X_{i},\alpha_{1}\right)\\ \left(1-Z_{i}\right)S_{0}\left(D_{i},X_{i},\alpha_{0}\right)\\ S_{\beta }\left(X_{i},\beta \right)\\ \left(\eta_{1}+\eta_{2}-\eta_{3}\right)/\left(\eta_{4}+\eta_{5}-\eta_{6}\right)-\mu_{j}^{c}\left(AIPW\right) \end{array}], \end{array}$$

where *S*_1_(*D*_*i*_, *X*_*i*_, *α*_1_) and *S*_0_(*D*_*i*_, *X*_*i*_, *α*_0_) are the likelihood score equations for *m*_1*i*_(*α*_1_) and *m*_0*i*_(*α*_0_), respectively. The last element of $\hat{\nu }$, obtained as a solution to $0=\sum_{i=1}^{N}\psi_{i}\left(\theta \right)$, is ${\hat{\mu }}_{j}^{c}\left(AIPW\right)$. Therefore, ${\hat{\mu }}_{j}^{c}\left(AIPW\right)$ is an M-estimator, which has an asymptotic normal distribution [25]. The variance of $\hat{\nu }$ is estimated by *N*^−1^*C*^−1^*DC*^−*T*^, with $C=-N^{-1}\sum_{i=1}^{N}\partial \psi_{i}{\left(\nu \right)/\partial \nu |}_{\nu =\hat{\nu }}$ and $D=N^{-1}\sum_{i=1}^{N}\psi_{i}\left(\nu \right)\psi_{i}{\left(\nu \right)}^{T}{|}_{\nu =\hat{\nu }}$. The estimated variance of ${\hat{\mu }}_{j}^{c}\left(AIPW\right)$ is the last diagonal element of *N*^−1^*C*^−1^*DC*^−*T*^. The asymptotic normal distributions of ${\hat{\mu }}_{j}^{a}\left(AIPW\right)$ and ${\hat{\mu }}_{j}^{n}\left(AIPW\right)$ can be demonstrated in a similar way.

## Supporting information

S1 Data (CSV)

## References

[1] Angrist JD, Imbens GW, Rubin DB. Identification of causal effects using instrumental variables. Journal of the American Statistical Association. 1996;91:444–55. doi: 10.1080/01621459.1996.10476902

[2] Marbach M, Hangartner D. Profiling Compliers and Noncompliers for Instrumental-Variable Analysis. Political Analysis. 2020;28(3):435–444. doi: 10.1017/pan.2019.48

[3] Abadie A. Semiparametric instrumental variable estimation of treatment response models. Journal of Econometrics. 2003;113:231–63. doi: 10.1016/S0304-4076(02)00201-4

[4] Card D. Using geographic variation in college proximity to estimate the return to schooling. In: Fagerberg J, Mowery DC, Nelson RR, editors. Aspects of Labour Market Behaviour: Essays in Honour of John Vanderkamp. Toronto: University of Toronto Press; 1995. p. 201–222.

[5] Baiocchi M, Cheng J, Small DS. Instrumental variable methods for causal inference. Statistics in Medicine. 2014;33(13):2297–2340. doi: 10.1002/sim.6128 24599889 PMC4201653

[6] Hangartner D, Marbach M, Henckel L, Maathuis MH, Kelz RR, Keele L. Profiling Compliers in Instrumental Variables Designs; 2021. Available from: https://arxiv.org/abs/2103.06328.

[7] Imbens GW, Angrist JD. Identification and estimation of local average treatment effects. Econometrica. 1994;62(2):467–475. doi: 10.2307/2951620

[8] Frölich M; Ctr Microdata Methods & Practice. Nonparametric IV estimation of local average treatment effects with covariates. Journal of Econometrics. 2007;139(1):35–75. doi: 10.1016/j.jeconom.2006.06.004

[9] Tan Z. Regression and weighting methods for causal inference using instrumental variables. Journal of the American Statistical Association. 2006;101(476):1607–1618. doi: 10.1198/016214505000001366

[10] Lunceford J, Davidian M. Stratification and weighting via the propensity score in estimation of causal treatment effects: a comparative study. Statistics in Medicine. 2004;23(19):2937–2960. doi: 10.1002/sim.1903 15351954

[11] Robins J, Rotnitzky A, Zhao L. Estimation of regression-coefficients when some regressors are not always observed. Journal of the American Statistical Association. 1994;89(427):846–866. doi: 10.1080/01621459.1994.10476818

[12] Marbach M. ivdesc: Profiling Compliers and Non-Compliers for Instrumental Variable Analysis; 2021. Available from: https://CRAN.R-project.org/package=ivdesc.

[13] Okui R, Small DS, Tan Z, Robins JM. Doubly robust instrumental variable regression. Statistica Sinica. 2012;22(1):173–205. doi: 10.5705/ss.2009.265

[14] Wang L, Tchetgen Tchetgen E. Bounded, efficient and multiply robust estimation of average treatment effects using instrumental variables. Journal of the Royal Statistical Society: Series B. 2018;80(3):531–550. doi: 10.1111/rssb.12262 30034269 PMC6051728

[15] Kang H, Jiang Y, Zhao Q, Small D. ivmodel: Statistical Inference and Sensitivity Analysis for Instrumental Variables Model; 2021. Available from: https://CRAN.R-project.org/package=ivmodel.

[16] An W, Wang X. LARF: Instrumental variable estimation of causal effects through local average response functions. Journal of Statistical Software. 2016;71(1):1–13.

[17] Rosenbaum P, Rubin D. The central role of the propensity score in observational studies for causal effects. Biometrika. 1983;70(1):41–55. doi: 10.1093/biomet/70.1.41

[18] Imai K, Ratkovic M. Covariate balancing propensity score. Journal of the Royal Statistical Society: Series B. 2014;76(1):243–263. doi: 10.1111/rssb.12027

[19] Choi BY, Wang CP, Michalek J, Gelfond J. Power comparison for propensity score methods. Computational Statistics. 2019;34:743–761. doi: 10.1007/s00180-018-0852-5

[20] Li F, Thomas LE, Li F. Addressing extreme propensity score via the overlap weights. American Journal of Epidemiology. 2019;188(1):250–257. 30189042 10.1093/aje/kwy201

[21] Wyss R, Ellis AR, Brookhart MA, Girman CJ, Funk MJ, LoCasale R, et al. The role of prediction modeling in propensity score estimation: an evaluation of logistic regression, bCART, and the covariate-balancing propensity score. American Journal of Epidemiology. 2014;180(6):645–655. doi: 10.1093/aje/kwu181 25143475 PMC4157700

[22] Tibshirani R. Regression Shrinkage and Selection via the Lasso. Journal of the Royal Statistical Society Series B (Methodological). 1996;58(1):267–288. doi: 10.1111/j.2517-6161.1996.tb02080.x

[23] van der Laan MJ, Polley EC, Hubbard AE. Super Learner. Statistical Applications in Genetics and Molecular Biology. 2007;6(1). doi: 10.2202/1544-6115.1309 17910531

[24] Choi BY, Wang CP, Gelfond J. Machine learning outcome regression improves doubly robust estimation of average causal effects. Pharmacoepidemiology and Drug Safety. 2020; p. 1–14. doi: 10.1002/pds.5074 32716126 PMC8098857

[25] Stefanski L, Boos D. The calculus of M-estimation. American Statistian. 2002;56(1):29–38. doi: 10.1198/000313002753631330

[26] Bang H, Robins JM. Doubly robust estimation in missing data and causal inference models. Biometrics. 2005;61(4):962–973. doi: 10.1111/j.1541-0420.2005.00377.x 16401269

[27] Tao Y, Fu H. Doubly robust estimation of the weighted average treatment effect for a target population. Statistics in Medicine. 2019;38:315–325. doi: 10.1002/sim.7980 30302780

